# A fast 3D reconstruction system with a low-cost camera accessory

**DOI:** 10.1038/srep10909

**Published:** 2015-06-09

**Authors:** Yiwei Zhang, Graham M. Gibson, Rebecca Hay, Richard W. Bowman, Miles J. Padgett, Matthew P. Edgar

**Affiliations:** 1 SUPA, School of Physics and Astronomy, University of Glasgow, Glasgow, G12 8QQ, UK; 2 Department of Physics, University of Cambridge, CB3 0HE, UK

## Abstract

Photometric stereo is a three dimensional (3D) imaging technique that uses multiple 2D images, obtained from a fixed camera perspective, with different illumination directions. Compared to other 3D imaging methods such as geometry modeling and 3D-scanning, it comes with a number of advantages, such as having a simple and efficient reconstruction routine. In this work, we describe a low-cost accessory to a commercial digital single-lens reflex (DSLR) camera system allowing fast reconstruction of 3D objects using photometric stereo. The accessory consists of four white LED lights fixed to the lens of a commercial DSLR camera and a USB programmable controller board to sequentially control the illumination. 3D images are derived for different objects with varying geometric complexity and results are presented, showing a typical height error of <3 mm for a 50 mm sized object.

Three dimensional (3D) image reconstruction is a procedure of creating a mathematical representation of a 3D object. 3D imaging has applications in a wide range of disciplines, for instance, prototyping, object recognition, robot navigation, 3D movies and games^1^.

There are various approaches for performing 3D imaging. On the smallest scale, interferometry is an extensively utilized technique for 3D surface reconstruction. The principle of interferometry is based on the phenomenon that two waves (e.g. light or radio waves) with the same or nearly the same frequency can be superimposed to form a resultant wave with a greater or lower amplitude^2^. With information extracted from the combined waves, interferometry can be used to inspect optical surfaces and provide high precision mapping to a small fraction of a wavelength^3^.

Another reliable non-contact 3D imaging technique is structured illumination. Having a calibrated projector-camera pair, a light pattern is projected onto the scene and imaged by the camera (or cameras). If the surface in the scene is planar without any 3D surface variation, the pattern shown in the corresponding image will be the same (or similar) to that of the projected light pattern. However, if the surface in the scene is non-planar, the projected structured-light pattern in the corresponding image will be distorted due to surface geometry^4^. The structured illumination method uses the information from the distortion of the projected structured-light pattern to extract the 3D surface geometry. By using various structured illumination patterns (most simply sine wave), 3D surface profiles of objects can be measured with a height error within millimeters^5^,^6^.

In addition to these aforementioned techniques, stereo vision (also known as stereoscopic vision or stereopsis) is another extensively used technique in 3D imaging, which reconstructs a 3D object by deducing the spatial shape and position of the object through parallax between the corresponding pixels from different images of the object as observed from multiple viewpoints^7^. The principle of traditional stereo vision techniques is triangulation, in which the unique contours of the object can be determined with the photos taken from two unparalleled cameras^8^. Traditional stereo vision approaches rely on the correspondence between photo elements from two cameras which sometimes can be difficult to determine.

It is also possible to extract 3D information from a single image, namely shape from shading^9^, by utilising assumptions of uniform surface reflectivity. More recently, there have been significant advances by using learning-based algorithms^10^, 3D morphable model algorithms^11^ and coupled radius basis function network algorithms^12^, that rely on large data training to recover 3D information from a single 2D image.

An alternative approach, which was first introduced by Woodham and known as photometric stereo^13^, allows depth and surface orientation to be estimated from multiple images of a static object taken from the same viewpoint, but under different illumination directions. This technique makes no assumption about the surface smoothness and can be calculated with reasonable computational cost.

There has been significant developments in the use of photometric stereo for 3D imaging in recent years. Okatani and Deguchi^14^ pointed out that, for general diffuse reflectance, there exists a set of the surface normals for which the relation between the surface normal and the orientation of the 3-vector formed by the image brightness triplet is guaranteed to be one-to-one. Basri, Jacobs and Kemelmacher^15^ demonstrated new methods of photometric stereo to recover surface normals assuming that all lights in a scene are distant from the object but otherwise unconstrained. Tan, *et al*.^16^ described a method to enhance the resolution of photometric stereo, which recovers the distribution of surface normals and the surface convexity of each pixel and then spatially arranging the normals among pixels based on the consistency and simplicity constraints on the surface structure. Kuparinen and Kyrki^17^ presented an optimal reconstruction of near planar textured surfaces using photometric stereo with the Wiener filtering from noisy and blurred observation, when the statistics of imaging errors are measurable. Shi, *et al*.^18^ proposed a self-calibrating method for photometric stereo, which uses color and intensity profiles from images taken under different and unknown lighting conditions to automatically recover both the camera’s radiometric response function and the unknown lighting directions and intensities. Hansen, *et al*.^19^ described an algorithm for selecting the optimal light sources used for photometric stereo reconstruction with both visible and near-infrared light sources, which does not require knowledge of the precise shadow boundary. Wu, *et al*.^20^ demonstrated a new approach for photometric stereo, which uses advanced convex optimization techniques to handle shadows and specularities in the images for recovering surface normals from multiple lighting conditions. Sun, *et al*.^21^ combined a lighting calibration method, which uses a reference face model to estimate the lighting parameters from face images taken under unknown illumination, with the classical photometric stereo to reconstruct 3D faces rapidly. Chandraker, *et al*.^22^ presented a comprehensive theory, in which surface information can be determined from unknown, isotropic bidirectional reflectance distribution functions (BRDFs). In subsequent work^23^, Chandraker demonstrated shape recovery from camera motions under BRDFs, based on shape from motion theories. Mecca, *et al*.^24^ investigated the implementations of photometric stereo in the case of near field imaging applications and presented an efficient model based on quasi-linear partial differential equations for surface reconstruction.

## Results

### Hardware system and image processing

**Figure 1:**
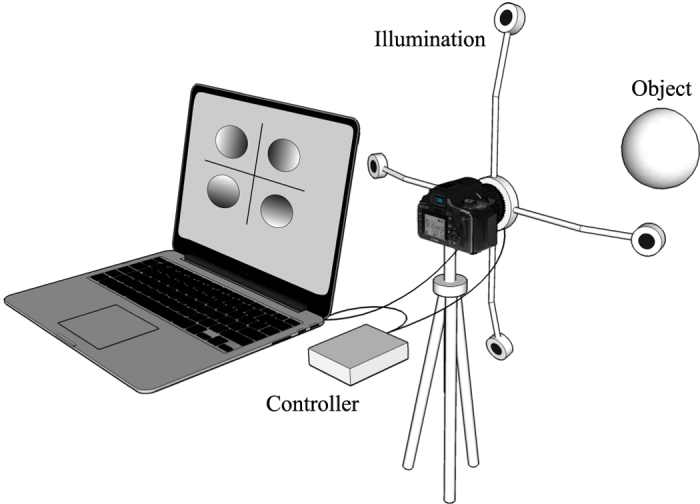
System setup. Schematic diagram (drawn by one of the co-authors) of system consisting of four LEDs, fixed around the camera lens and connected to a electronic controller board. The object was placed normal to the camera lens. Both camera and controller board were controlled by the program on the laptop.

Our system consists of a commercial DSLR camera (Canon EOS 5D MarkII), four white LEDs (Luxeon Rebel) surrounding the camera lens, fixed at a distance of 330 mm via aluminium spokes, a controller board (Arduino Uno) to enable USB control of the illumination direction, and a computer running our program (LabVIEW) to communicate with the controller board and obtain real-time 2D images captured by the camera (see Fig. 1).

**Figure 2:**
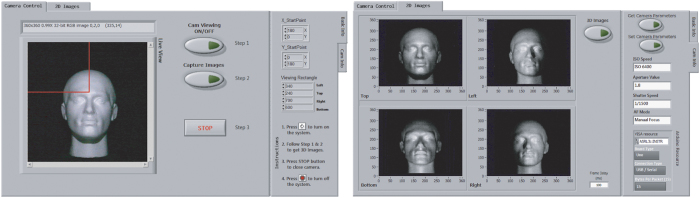
Program interface. The Camera Control tab displays the live view from the camera with a manually cropped area. The red cross point in the viewing area is the set start point of reconstruction. The 2D Images tab shows the four images captured under various lighting directions. The Cam Info tab contains the camera parameter settings.

For this investigation the background of each scene is set to be black, which helps objects to be segmented from it. The object position, the camera perspective, and the light positions for all images are static and known. Once the object and camera are aligned, image acquisition is triggered through our software (see Fig. 2), initiating four LED’s to flash successively, synchronized with a short camera exposure (in total less than 1 second). This procedure provides four separate images of the object, each with shading determined by a different lighting vector. To minimize computer memory requirements, the images are subsequently resized and down-sampled to 360 × 360 pixels before 3D image reconstruction is performed.

**Figure 3:**
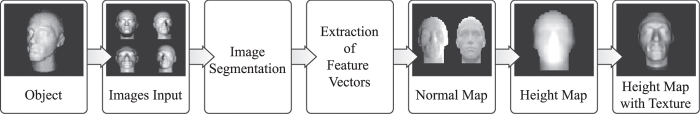
Reconstruction program pipeline. The reconstruction program executes following the sequence: image input, image segmentation, extraction of feature vectors, normal map, height map, and height map with texture.

The software reconstruction pipeline is shown in Fig. 3. The intensities from the four 2D input images are compared and the maximum intensity is obtained to provide the red, green and blue (RGB) intensity values for each pixel. This provides the reflection coefficient *ρ*^*t*^ map, which is later used for the 3D object texture. A threshold value is set to distinguish background and foreground, from which the object in the images can be separated. With the known coordinates, *L*_*n*_, of four LED’s and the corresponding object intensity value on each pixel *I*_*n*_, the object’s surface normal *N* is calculated by rearranging^25^


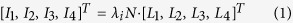


where λ_*i*_ is the albedo (reflectivity) of each pixel on the object surface that can be estimated with a modular operation of the surface normal.

From the surface normal of each pixel, the gradients between adjacent pixels are used to obtain the surface geometry by integration, from a central starting point (set manually) to the outermost pixels of the object’s surface. The surface height at a pixel point can be approximated with the gradient of the surface and the height of its nearest-neighbor point. Since each pixel point corresponds to the measured gradient data and has more than one nearest-neighbor point, the gradient of the surface used is the mean value of the gradients at every two contiguous points, and the surface height at each pixel point is denoted by the mean of the values calculated from all the nearest-neighbor points.

The program runs through the pixel points one at a time, each time the height at the pixel point in each iteration is set to the mean value of its estimates from all the nearest-neighbor points. The pixel points of an object can be divided into two types: internal pixel point and boundary pixel point. When a pixel is an internal point, the reconstructed height field’s Laplacian is set to the Laplacian calculated from the measured gradient data; when a pixel is a boundary point, the measured gradient data at the point is assumed to be accurate^26^.

### Quantitative and qualitative comparison

**Figure 4:**
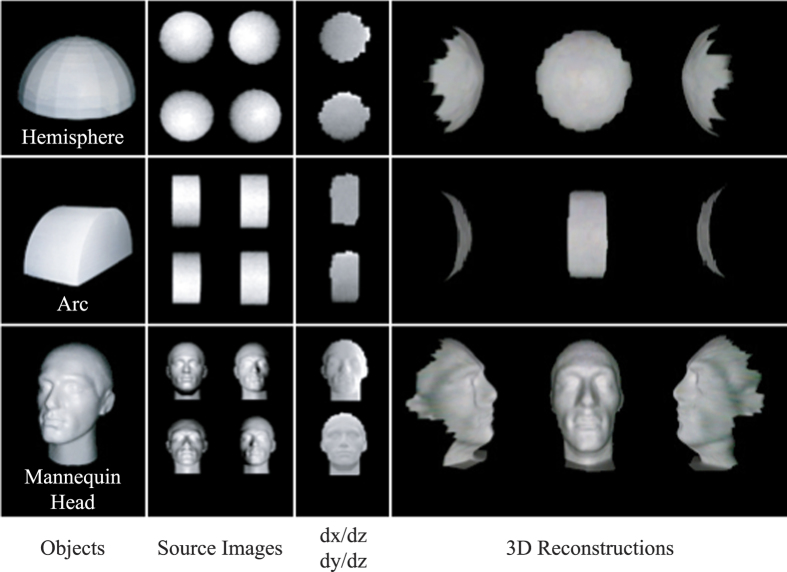
Reconstruction results of objects: a hemisphere, an arc, and a mannequin head. From left to right: object images, images captured with four different illumination directions, gradient maps *dx*/*dz* and *dy*/*dz*, 3D reconstructions from left, center, and right directions.

To test the accuracy and robustness of our 3D imaging system we imaged three different objects with varying geometric complexity: a hemisphere, an arc, and a mannequin head. Both the hemisphere and the arc were constructed using a 3D printer and designed with a height of 50 mm, while the mannequin head was measured to have a height (from ear to nose tip) of 160 mm. The front of each object was located 900 mm from the camera lens. Surrounding the lens were located four white LED’s (positioned above, below, left, and right) maintained at a distance of 330 mm from the center of the lens. For each of the objects placed in the scene, four images were acquired, corresponding to the different lighting conditions and cropped into a 360 × 360 pixel image (corresponding to 320 mm × 320 mm virtual size). These images were then used to reconstruct the 3D surface of the objects (see Fig. 4) using the aforementioned approach.

The 3D surface height map for each object was compared with a reference height map. For the arc and the hemisphere, the reference data was acquired from a stereolithography (STL) file used to create them, whereas the mannequin head reference data was obtained from a stereophotogrammetric camera system. For comparison, the measured data was scaled appropriately to match the reference data. We express the standard deviation of the differences between measured data and reference data using the root mean square error (RMSE) and the normalized root mean square error (NRMSE), representing the variation of the RMSE^27^. The RMSE and NRMSE, correspondingly are defined as^28^:


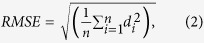



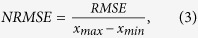


where *n* is the number of data pairs, *d*_*i*_ is the difference between measured values and reference values, and (*x*_*max*_ − *x*_*min*_) is the range of measured values. The value of NRMSE is often expressed as a percentage, where a lower value indicates less variance and hence higher accuracy. The results are shown in Table 1.

**Figure 5:**
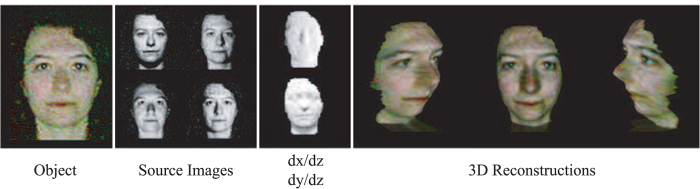
3D image reconstruction of a real human subject.

We observed close agreement for the two objects with relatively low geometric complexity, the hemisphere and the arc, having RMS errors of 2.76 mm and 2.65 mm, respectively. While an RMS error of 15.60 mm was observed for the mannequin head. We note that the regions contributing most to the overall RMS error were locations of sharp edges or where the surface normal was in a direction perpendicular to the camera perspective. Furthermore, we tested the system with a real human subject, to validate its capabilities for potential applications, such as facial recognition, the result of which is shown in Fig. 5.

In addition to reconstructing 3D images with a cheap camera attachment and photometric stereo software, it is possible to simultaneously determine pairs of 2D images representing the slightly different perspectives of an object that would be formed by our eyes. By displaying these images on a 3D enabled TV, we are able to view the object in 3D from different angles despite the camera and object remaining stationary during image acquisition. We have demonstrated this system in application whilst obtaining 3D images of faces from visitors at various science exhibitions (such as the Glasgow Science Centre and the Royal Society), whereupon a short 2D movie is produced of the head rotating and subsequently sent to an email address. The results proved the capability of this system to reconstruct 3D models of faces under realistic workplace conditions.

## Discussion

In this paper, we have presented a new computational system with a low-cost camera accessory, which consists of four white LED’s and a controller board, for enabling fast 3D image reconstruction based on photometric stereo principles. We have performed 3D reconstructions on a selection of objects with varying geometric complexity, finding good quantitative agreement with the known reference object with a wide viewing angle. We observed an increased error at regions of large gradients, where the surface normals were approaching a direction perpendicular to the camera perspective, indicating one limitation of this 3D imaging system. This inexpensive system has potential to be used at the airport security-check for collecting 3D information of passengers rapidly, and it could also be applied to high schools for education purpose. Further improvement could be made to the system by optimising the reconstruction algorithm in order to provide better height estimates at regions with sharp edges.

## Methods

### Photometric Stereo

**Figure 6:**
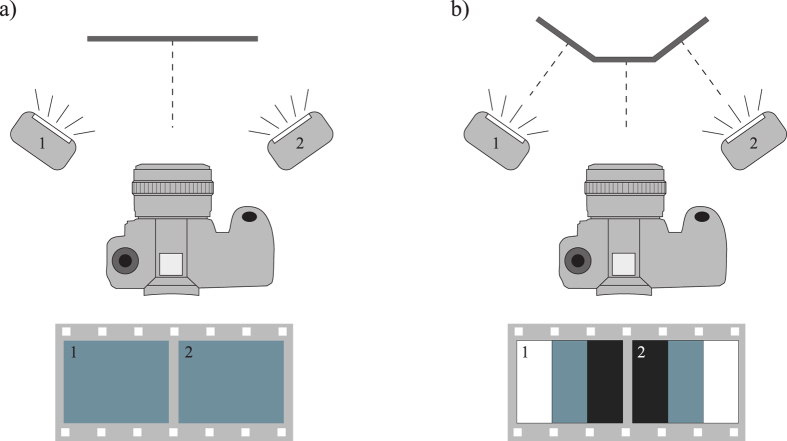
Photometric stereo. **a**) When the surface of an object is flat and normal to the camera lens, images taken using axis lighting directions 1 and 2 will have the same light intensity of the object. **b**) When the surface of an object is uneven, images taken using lighting directions 1 and 2 will have different light intensities.

The appearance of an object in a photo results from the effects of illumination, object orientation, object shape and its reflectance. With a fixed object, the corresponding surface orientation can be determined by analyzing the object images under different illumination directions (see Fig. 6). Fundamentally, photometric stereo, which is simple and succinct for Lambertian surfaces, enables 3D reconstruction of an object by analyzing differences in the pixel intensities in images that have been acquired from at least three different illumination directions^29^.

By definition, a surface, from which light is reflected in all directions and its brightness looks the same regardless of direction and position, is a Lambertian surface^30^. For instance, a human face is an approximate Lambertian object^31^, for which this system may have applications in imaging, for example in facial recognition techniques. In general, the appearance of a diffuse object with a specular varying reflection may be modeled as^32^:


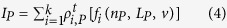


where *I*_*P*_ is the pixel intensity at point p, *k* is a fixed value of a linear combination of *k* basis materials, *ρ*^*t*^ is a reflection coefficient that varies on the surface, *f*_*i*_ is any reflectance map as a function of the viewing direction *v*, *n*_*P*_ is the surface normal at that point, and *L*_*P*_ is the incident illumination field.

Photometric stereo requires some control of the lighting environment, without position changes of neither object nor camera. Theoretically, three illumination directions are sufficient to obtain the surface normals, however to ensure that at least three intensity values are measured at any pixel in all acquired images, our 3D imaging system utilizes four illumination directions.

The data used to produce the content of this manuscript is available at: http://dx.doi.org/10.5525/gla.researchdata.168.

## Additional Information

**How to cite this article**: Zhang, Y. *et al*. A fast 3D reconstruction system with a low-cost camera accessory. *Sci. Rep*. **5**, 10909; doi: 10.1038/srep10909 (2015).

## Supplementary Material

Supplementary Information

Supplementary Movie S1

Supplementary Movie S2

## Figures and Tables

**Table 1:** Deviations between measured values and true values.

Object	Height (mm)	Scale Factor	RMSE (mm)	NRMSE
Hemisphere	50	2.66	2.76	5.53%
Arc	50	3.14	2.65	5.32%
Head	160	2.02	15.60	9.76%
