# Supplementary material for: A fast 3D reconstruction system with a low-cost camera accessory
Source: Sci Rep. 2015 Jun 9;5:10909. doi: 10.1038/srep10909 (PMC4460880; doi:10.1038/srep10909)
Supplement: Supplementary Information [file srep10909-s1.pdf]

Supplementary Information for

A fast 3D reconstruction system with a low-cost camera accessory

Yiwei Zhang*, Graham Gibson, Rebecca Hay, Richard Bowman,

Miles J. Padgett, and Matthew P. Edgar

*Correspondence to: EwayZhang@gmail.com

This Supplementary Information includes:

Captions for videos 1-2

Captions for videos 1-2

Video 1: 3D video reconstruction (high-resolution) of a mannequin head artificially rotated from left to right. The time for 2D image capture, 3D image reconstruction and output to 3D enabled TV is less than 10s.

Video 2: 3D video reconstruction (low-resolution) of a head from a human subject, artificially rotated from left to right. The time for 2D image capture, 3D image reconstruction and output to 3D enabled TV is less than 10s.
